# Cryptococcal infection with ruxolitinib in primary myelofibrosis: A case report and literature review

**DOI:** 10.1002/ccr3.5461

**Published:** 2022-02-20

**Authors:** Zachary Ciochetto, Njeri Wainaina, Mary Beth Graham, Anna Corey, Muhammad Bilal Abid

**Affiliations:** ^1^ 5506 Division of Infectious Diseases Medical College of Wisconsin Milwaukee Wisconsin USA

**Keywords:** *Cryptococcus neoformans*, invasive fungal infection, myeloproliferative neoplasm, primary myelofibrosis, ruxolitinib

## Abstract

Cryptococcus neoformans (CN) is an encapsulated yeast that is found worldwide. It causes self‐limiting infections in immunocompetent hosts; however, infections due to CN could be disseminated and potentially life‐threatening in immunocompromised hosts. Herein, we present a patient with primary myelofibrosis who received ruxolitinib and developed disseminated cryptococcosis due to CN. We further discuss immune compromising factors indigenous to myeloproliferative neoplasms, ruxolitinib, and immunological pathways associated with janus kinase inhibition. We further review other cases of cryptococcal infections in patients receiving ruxolitinib reported in the literature. The report underscores the importance of suspecting infections with intracellular pathogens early in the course of illness in patients with higher rates of cumulative immunosuppression. A high clinical suspicion should be maintained when caring for such immunosuppressed patients receiving immunomodulatory agents as severe, disseminated infections can present atypically and lead to worse outcomes.

## 1 INTRODUCTION

The Cryptococcus species complex are encapsulated yeasts comprising two pathogenic species, *Cryptococcus neoformans* (CN) and *Cryptococcus gattii* (CG). CN is distributed globally in soil, particularly when enriched with bird droppings, while CG is endemic to tropical and sub‐tropical climates. Cryptococcosis is acquired via inhalation of the spores from the soil into the host's lungs leading to an asymptomatic or mild self‐limiting infection if immunocompetent. In contrast, in immunocompromised individuals, it can lead to severe primary or reactivated disease. The highest morbidity and mortality are seen with dissemination to the central nervous system causing meningitis, but it can also involve the skin, bone, spleen, and other tissues.^1^

Cryptococcosis is an acquired immunodeficiency syndrome (AIDS) defining infection and is also associated with conditions that disrupt effector T‐cell function such as solid organ and hematopoietic cell transplantation, progressive cancers, and prolonged corticosteroid use. The hosts’ innate and adaptive immune pathways defend against cryptococcal infection, but the fungus attempts to evade the immune system using several mechanisms including its polysaccharide capsule.^2^ Th1 and Th2 cells, along with cytokines, directly activate CD8+ and natural killer (NK) cells which perform the fungicidal activity through perforins and granzymes.^2^ Hosts with functional or numeric decline in T cells develop disseminated infection as they cannot mount adequate immune response.

Myeloproliferative neoplasms (MPNs) are stem cell‐derived clonal myeloid malignancies characterized by a unique somatic mutational profile with three common mutations (JAK2, MPL, and CALR). These mutations account for 90% of cases, whereas 10% of patients do not have any of the three mutations listed. These driver mutations, if present, result in constitutive activation of Janus kinase (JAK2)‐dependent signaling.

Primary myelofibrosis (PMF) is a variant of MPNs that develops as fibrotic material replaces the bone marrow, resulting in extramedullary hematopoiesis. Ruxolitinib is a selective oral inhibitor of both JAK1/2 protein kinases. Its use in patients with PMF is associated with a reduction in spleen volume as well as improvement in symptoms and survival. Evidence shows that ruxolitinib exerts potent anti‐inflammatory and immunosuppressive effects. Cellular targets of ruxolitinib include components of both the innate and adaptive immune system, such as NK and DCs, helper T cells, and regulatory T cells (Tregs).^3^

Herein, we present a patient who developed disseminated cryptococcosis while receiving ruxolitinib therapy for PMF. We further present relevant literature and discuss immunologic mechanisms involved in the fungal pathogenesis in immunocompromised patients. A review of 5 other cryptococcal infections in patients on ruxolitinib was performed (Table 1), further highlighting various disease presentations, pathology, and characteristics.^11^, ^12^, ^13^, ^14^, ^15^ Immunocompromised patients’ immune suppression can be further exacerbated by immunomodulating treatments; thus, clinicians must remain vigilant in looking for signs of infection due to intracellular pathogens.

**TABLE 1:** Patient, disease, and treatment characteristics of patients receiving ruxolitinib for MPN

Reference	Type of infection	Age, years	Sex	Country	Days after ruxolitinib initiation	Type of MPN	Ruxolitinib discontinued	Duration of Ruxolitinib	Prophylaxis (pre‐infection)	Treatment of CN (agent and duration)	Outcome
Wysham 2013	Cryptococcus neoformans pneumonia	66	M	USA	18 months	MF, post‐PCV	Yes	18 months	No	Fluconazole 400 mg, 5 months	Survived
Chen 2016	Cryptococcal meningoencephalitis	69	F	Taiwan	3 years 10 months	Myelofibrosis	NR	4 years	No	Fluconazole 400 mg, indefinitely	Survived
Hirano 2017	Pulmonary cryptococcosis	79	M	Japan	6 months	Primary Myelofibrosis	Yes	6 months	No	Voriconazole 400 mg daily, 3 months	Survived
Tsukui 2020	Cryptococcal meningitis	72	M	Japan	5 months	Myelofibrosis	Yes	5 months	No	37 days LAP followed by indefinite Fluconazole 400 mg daily	Survived
Prakash 2019	Cryptococcal meningitis	51	M	Guam	18 months	Polycythemia vera	Yes	18 months	No	3 months LAB followed by indefinite Isavuconazole 372 mg daily	Survived

Abbreviations: CN, cryptococcus neoformans; LAP, liposomal amphotericin‐B; MF, myelofibrosis; MPN, myeloproliferative neoplasm; PCV, polycythemia vera; PMF, primary myelofibrosis; USA, United States of American.

## 2 CASE PRESENTATION

An 82‐year‐old man with a past medical history of PMF was admitted to the hospital with headache, nausea, and vomiting for 5 days. Five years prior, he had been diagnosed with high‐risk PMF with severe reticulin fibrosis on bone marrow morphology and positive JAK2 and CALR mutations based on genetic analysis. He received hydroxyurea initially for one year and then received low‐dose corticosteroids and ruxolitinib due to worsening anemia, thrombocytopenia, leukocytosis, and splenomegaly. In the emergency department, he was afebrile, blood pressure was 176/106 mmHg, and pulse was 51 beats per minute, with normal oxygenation and respiratory rate. He was alert and oriented, and there were no cognitive or focal neurological deficits. The patient did not exhibit nuchal rigidity, lymphadenopathy, jaundice, icterus, or hepatomegaly. Physical examination was unremarkable other than mild non‐tender splenomegaly.

Initial laboratory testing was notable for sodium of 125 mEq/L (normal 135–145 mEq/L), serum osmolality 260 mOsm/kg (normal 285–295 mOsm/kg), lactate dehydrogenase (LDH) 974 unit/L (normal 135–225 unit/L), hemoglobin 7.7 g/dL (normal male 13.5–17.5 g/dL), and thrombocytopenia at 69 × 10^9^/L (normal 150–450 × 10^9^/L). His anemia and thrombocytopenia were at his known baseline levels. His white blood cell (WBC) count was 32.9 × 10^9^/L (normal 4–12 × 10^9^/L) with 35% of the differential comprised of myelocytes and 6% blasts. Head and contrasted visceral imaging were unremarkable, and blood cultures were obtained.

The patient developed seizures 5 days into the treatment for his syndrome of inappropriate antidiuretic hormone, prompting further investigation. Computerized tomography (CT) and magnetic resonance imaging (MRI) of the patients' head were negative, along with unremarkable electroencephalography (EEG). CT chest revealed bilateral pulmonary nodules. Blood cultures grew yeast two days after acquisition, so intravenous (IV) micafungin 100mg daily was initiated, and a serum cryptococcal antigen was tested. On the 7th day of hospitalization, the yeast in blood cultures was identified as CN, and the serum cryptococcal antigen returned positive with titers of 1:>1024 (normal <1:2). In consultation with infectious diseases, IV liposomal amphotericin‐B 3 mg/kg daily and flucytosine 25mg/kg twice daily was initiated and a lumbar puncture recommended. Unfortunately, the patient succumbed before the suggested diagnostics and therapeutics could be performed.

## 3 DISCUSSION

We present a patient who developed irreversible sepsis from suspected disseminated cryptococcal infection due to CN temporally associated with the use of ruxolitinib for PMF. Cell‐mediated immunity is essential in preventing severe infection with intracellular pathogens such as CN. The case underpins the immunological principle of compounding immunosuppression.

In PMF with dysregulated JAK1/2 pathways, myeloid cells show altered expression of both chemokine and cytokine receptors along with reduced ability to produce inflammatory cytokines in response to infectious stimuli. This phenomenon reduces cell proliferation and migration to infected tissues with reduced activation of NK cells, impairing the hosts’ defenses. This can be compounded by ruxolitinib inhibition of interleukin (IL)‐4, which negatively effects downstream signaling and differentiation of monocytes into NK cells. This compounding immunosuppression thus increases the hosts risk for infections.^4^, ^5^

Treatment of PMF with ruxolitinib acts by inhibiting the JAK1/2 pathways, which prevents abnormal hematopoiesis.^6^, ^7^ Concurrently, modulation of critical JAK pathways and downstream signaling compromises both innate and adaptive immune systems, predisposing patients to bacterial, viral, and fungal infections, which are the main cause of morbidity and mortality in patients with PMF.^8^ The higher the International Prognostic Scoring System (IPSS) and greater the spleen volume, the higher the risk of infection.^9^, ^10^ Pivotal clinical trials (COMFORT‐I, COMFORT‐II, and JUMP) showed that patients with PMF treated with ruxolitinib had reduction in spleen size, decreased constitutional symptoms, and improved survival.^14^, ^15^, ^16^

The COMFORT‐I and COMFORT‐II trials also reported an increased risk of bacterial and viral infections in patients treated with ruxolitinib.^14^, ^15^ COMFORT‐II reported a higher incidence of herpes zoster infections (11.5% of patients), pneumonia (13.1%), sepsis (7.6%), and urinary tract infections (24.6%), with two cases of tuberculosis. It was recognized that myeloperoxidase deficiency was seen with homozygous CALR mutations, leading to neutrophil dysfunction. With diminished functional efficacy of neutrophils, DCs, NK cells, and Th1 and Th17 cells, patients are at a higher risk for disseminated viral and fungal infections.

Additionally, we performed a literature review of invasive cryptococcal infections associated with immunosuppressive agents such as ruxolitinib. Two published reports of cryptococcal pulmonary infection and 3 of cryptococcal meningitis are presented in Table 1.^11^, ^12^, ^13^, ^14^, ^15^ A cohort of 65 patients on treatment with ruxolitinib for either polycythemia vera, primary or secondary myelofibrosis, saw 2 patients develop disseminated mycobacterial infection. One case was due to *Mycobacterium tuberculosis* and the other *Mycobacterium avium complex*, suggesting ruxolitinib predisposes patients to disseminated mycobacterial infections more than previously observed.^17^ Our patient, along with other cases in Table 1, highlights the need for increased surveillance for intracellular pathogens, continued antimicrobial prophylaxis, and high clinical suspicion for fungal infections in patients with PMF receiving ruxolitinib. Providers caring for patients with MPNs receiving ruxolitinib need to pursue a personalized risk‐adapted approach in gauging ruxolitinib's morbidity and mortality benefits against the risks for opportunistic infections. Further data on epidemiology and severity of infections seen with ruxolitinib use in patients with MPNs are needed.

This manuscript summarizes the immunological processes of PMF and the immunomodulatory impact of JAK pathway inhibitors. Both factors cumulatively lead to patients' susceptibility to cryptococcosis. This fungal infection can quickly lead to disseminated disease with high morbidity and mortality in case of delay in diagnosis suspected in immunocompromised individuals. There are few case reports described in the literature of cryptococcal infection on ruxolitinib therapy. Our report adds to the current data and underscores the immune dysregulation associated with the underlying disease and the treatment. The case report with the literature review highlights for further studies examining the immune mechanisms that predispose patients to potentially fatal infections and abrogate the remission success.

## CONFLICT OF INTEREST

None.

## AUTHOR CONTRIBUTIONS

ZC put together the manuscript with the help of MBA. Manuscript was reviewed and edited with the help of NW and MBG.

## ETHICS APPROVAL

None.

## CONSENT

Written informed consent was obtained from the patient for publication of this case report and any accompanying images. A copy of the written consent is available for review by the Editor of this journal.

## Data Availability

None.
